# Supplementary figures and images for: Stabilization of interdependent networks with two sub-networks of non-identical nodes
Source: PLoS One. 2025 Dec 26;20(12):e0337899. doi: 10.1371/journal.pone.0337899 (PMC12742803; doi:10.1371/journal.pone.0337899)

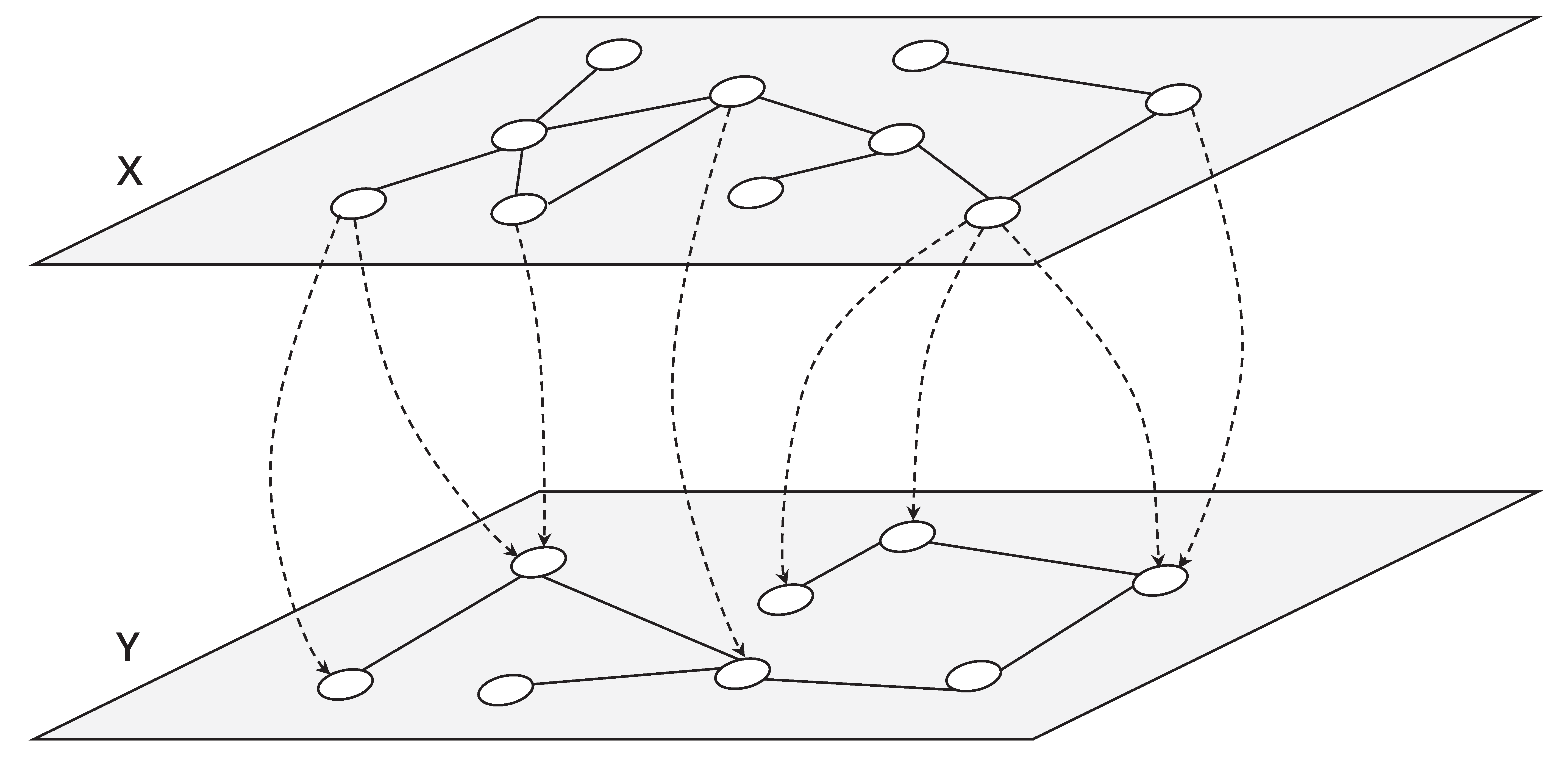

Supplement: S1 File — (TIFF) [file pone.0337899.s001.tif]

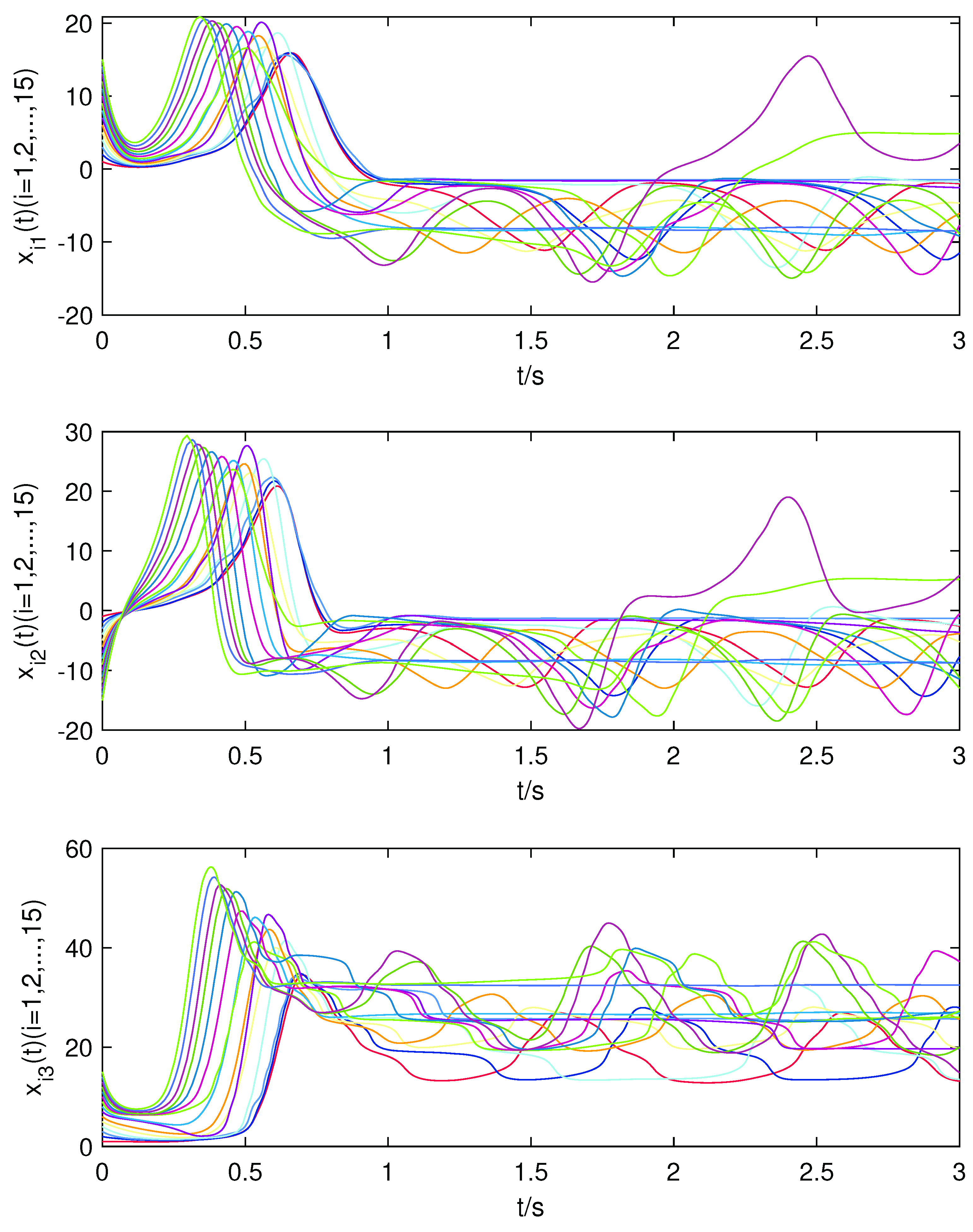

Supplement: S2 Fig — (TIFF) [file pone.0337899.s002.tif]

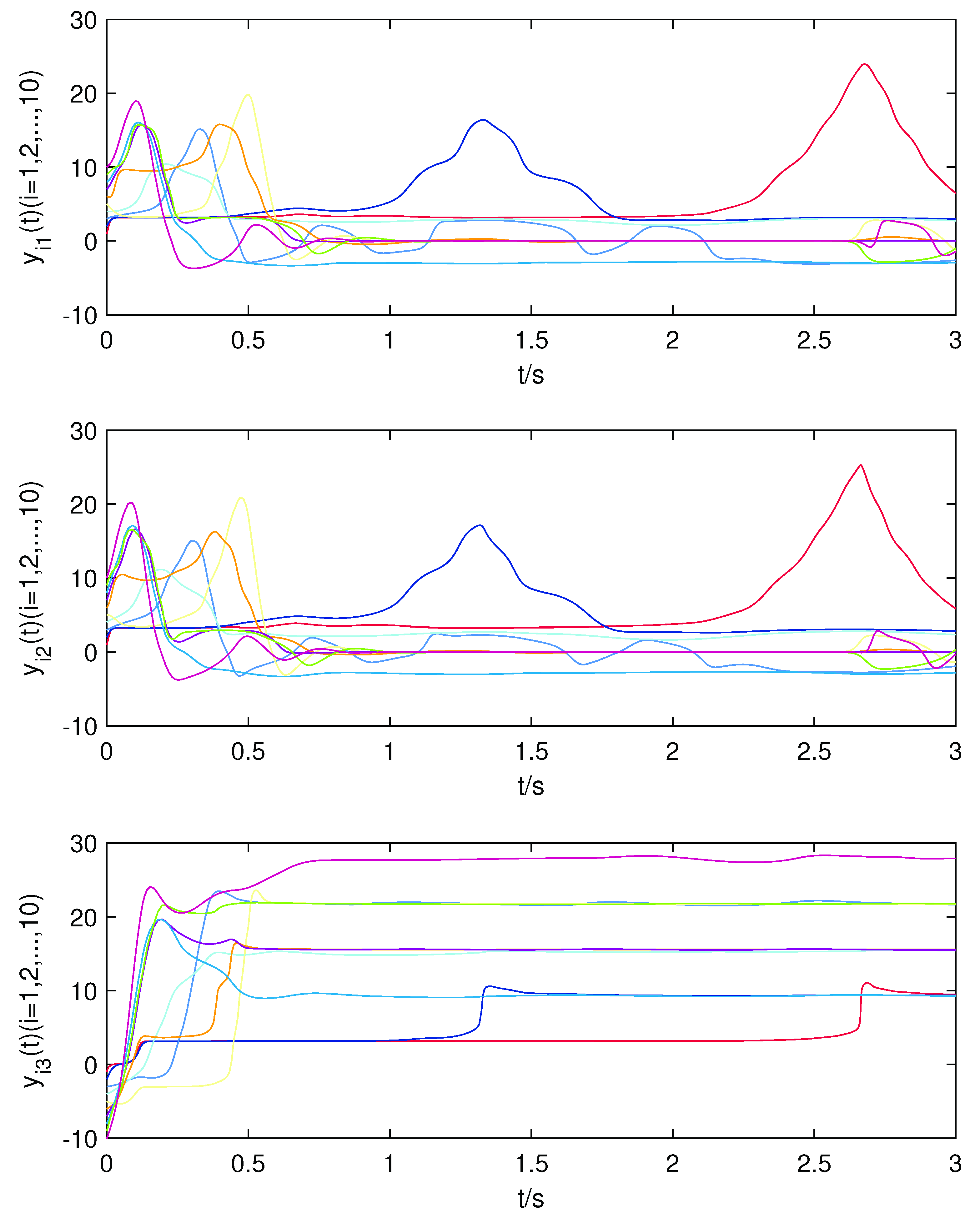

Supplement: S3 Fig — (TIFF) [file pone.0337899.s003.tif]

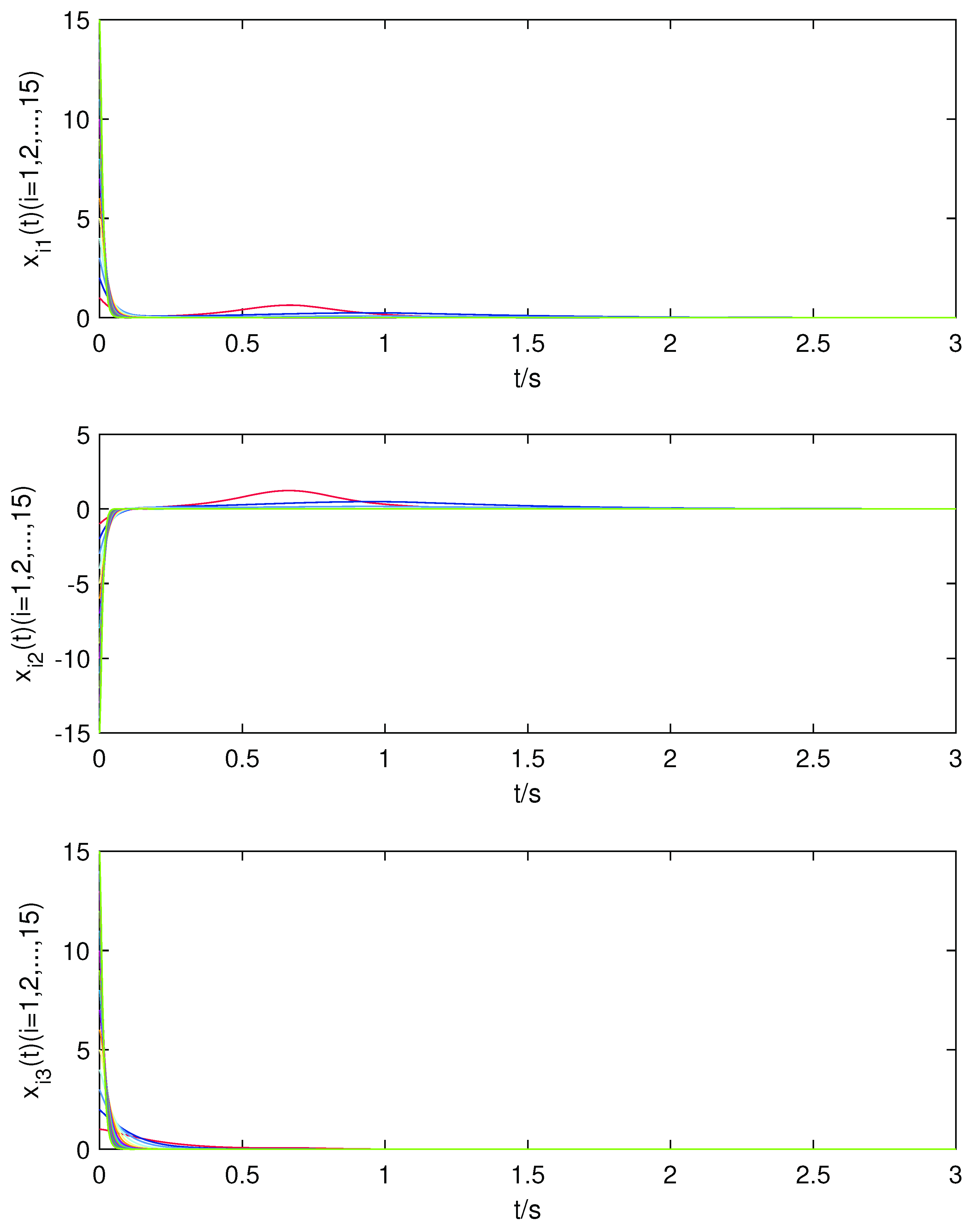

Supplement: S4 Fig — (TIFF) [file pone.0337899.s004.tif]

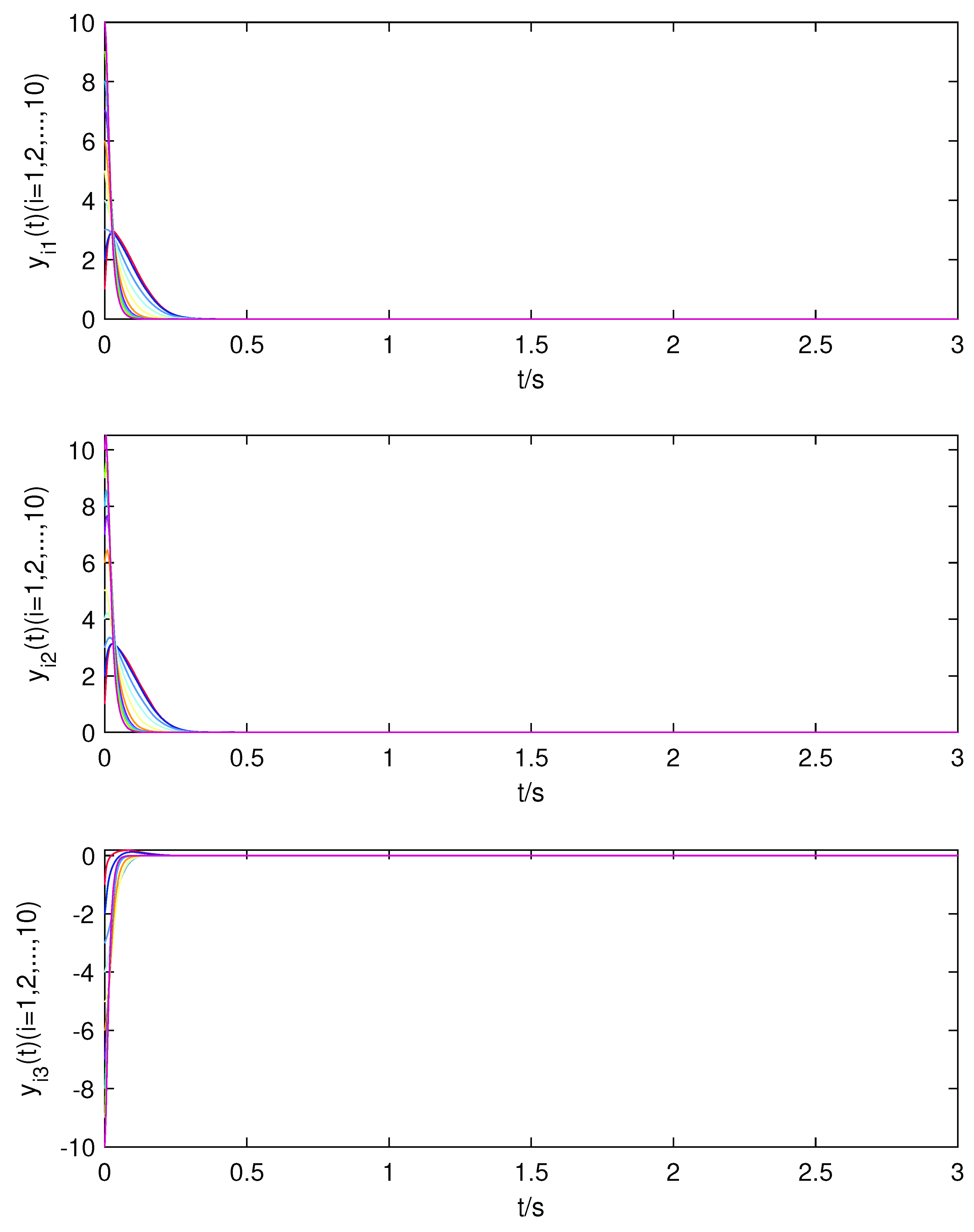

Supplement: S5 Fig — (TIFF) [file pone.0337899.s005.tif]

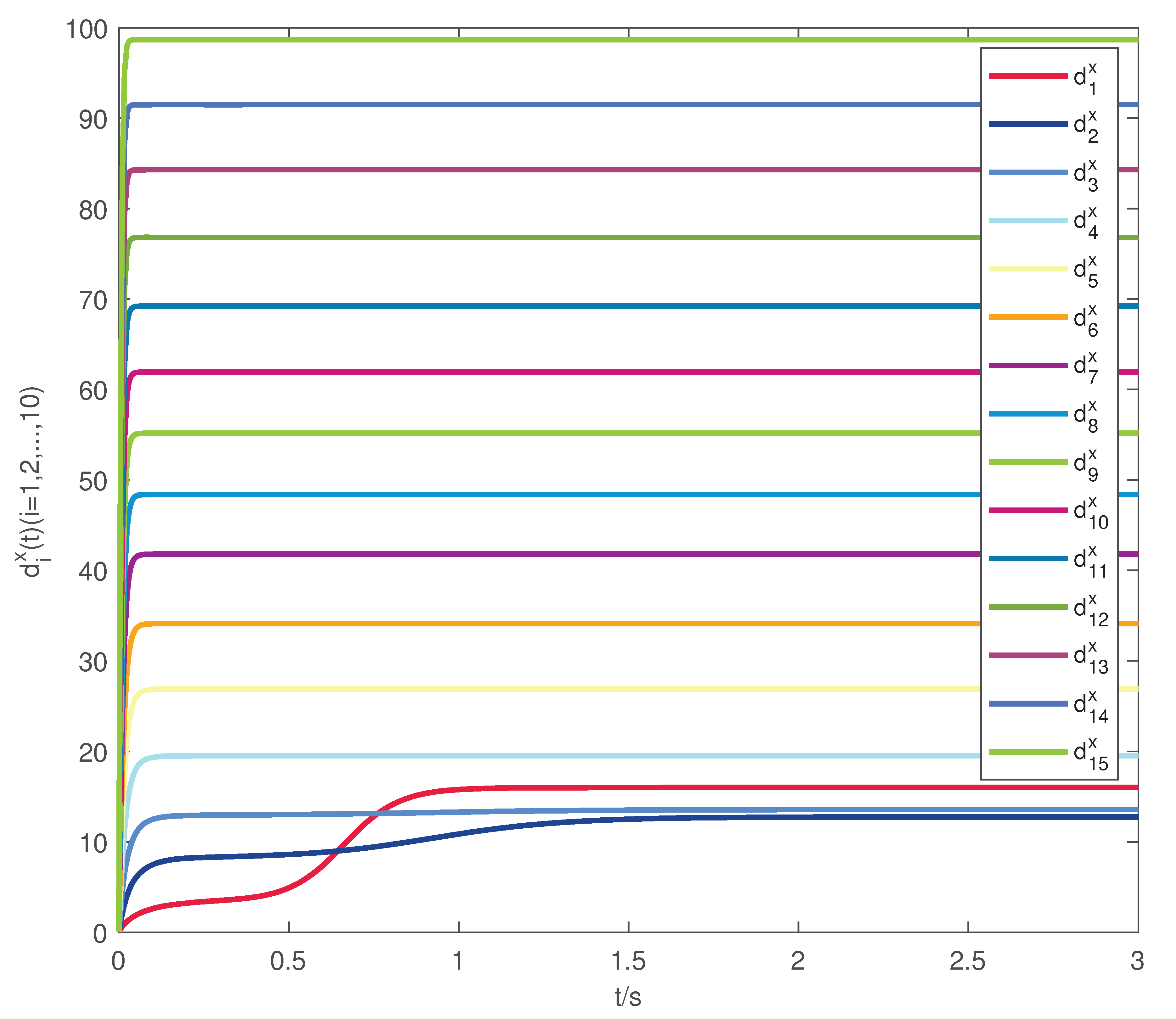

Supplement: S6 Fig — (TIFF) [file pone.0337899.s006.tif]

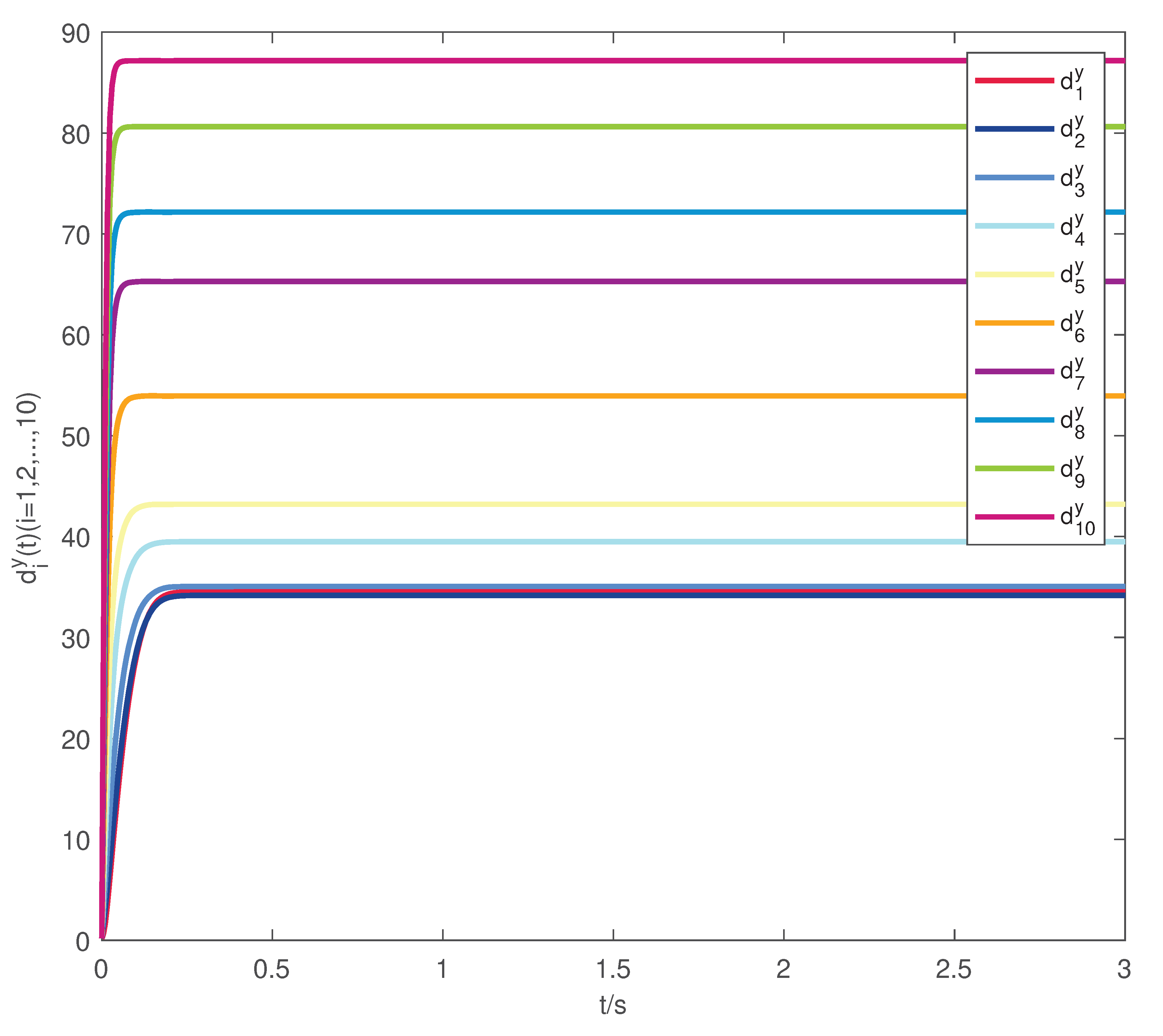

Supplement: S7 Fig — (TIFF) [file pone.0337899.s007.tif]

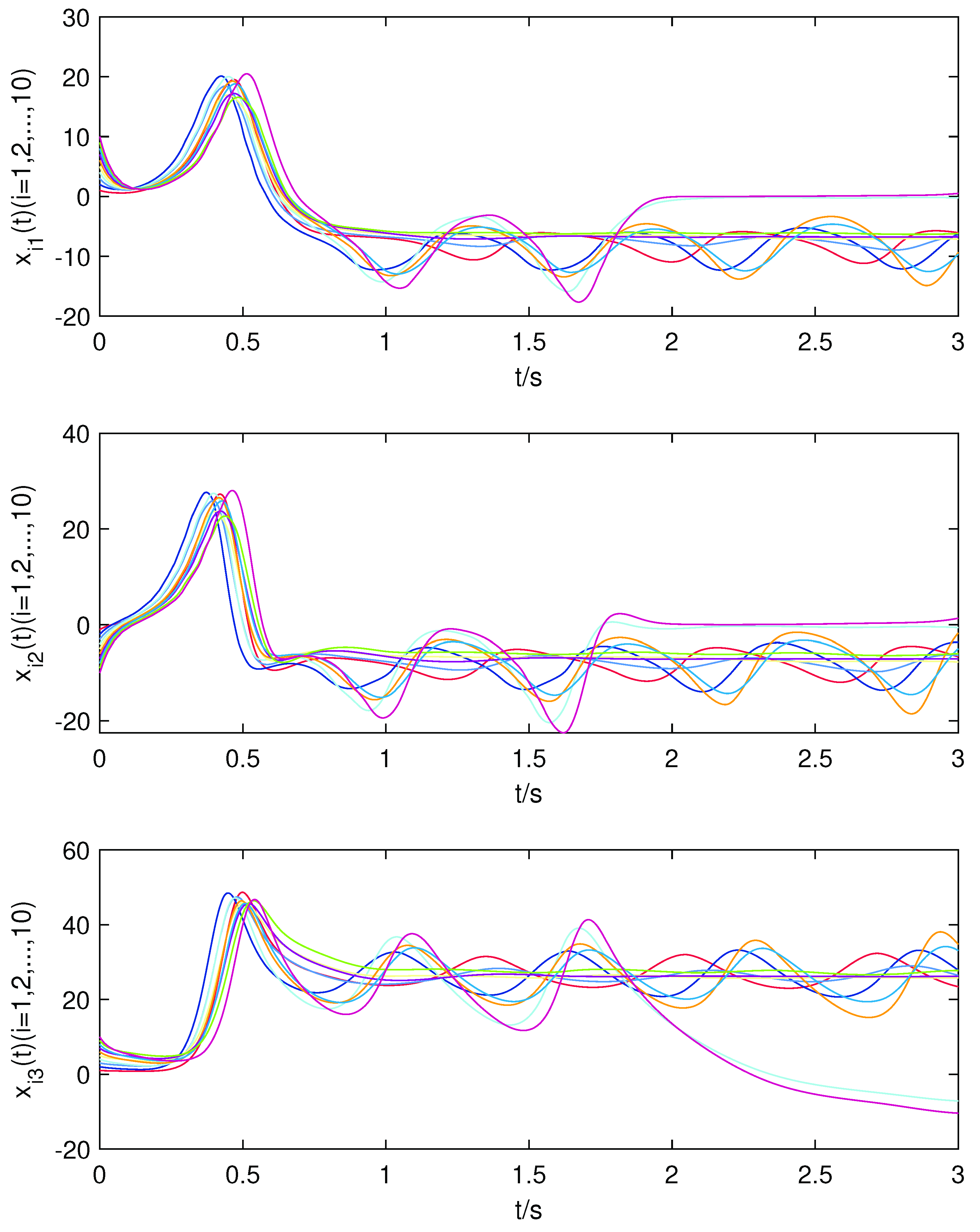

Supplement: S8 Fig — (TIFF) [file pone.0337899.s008.tif]

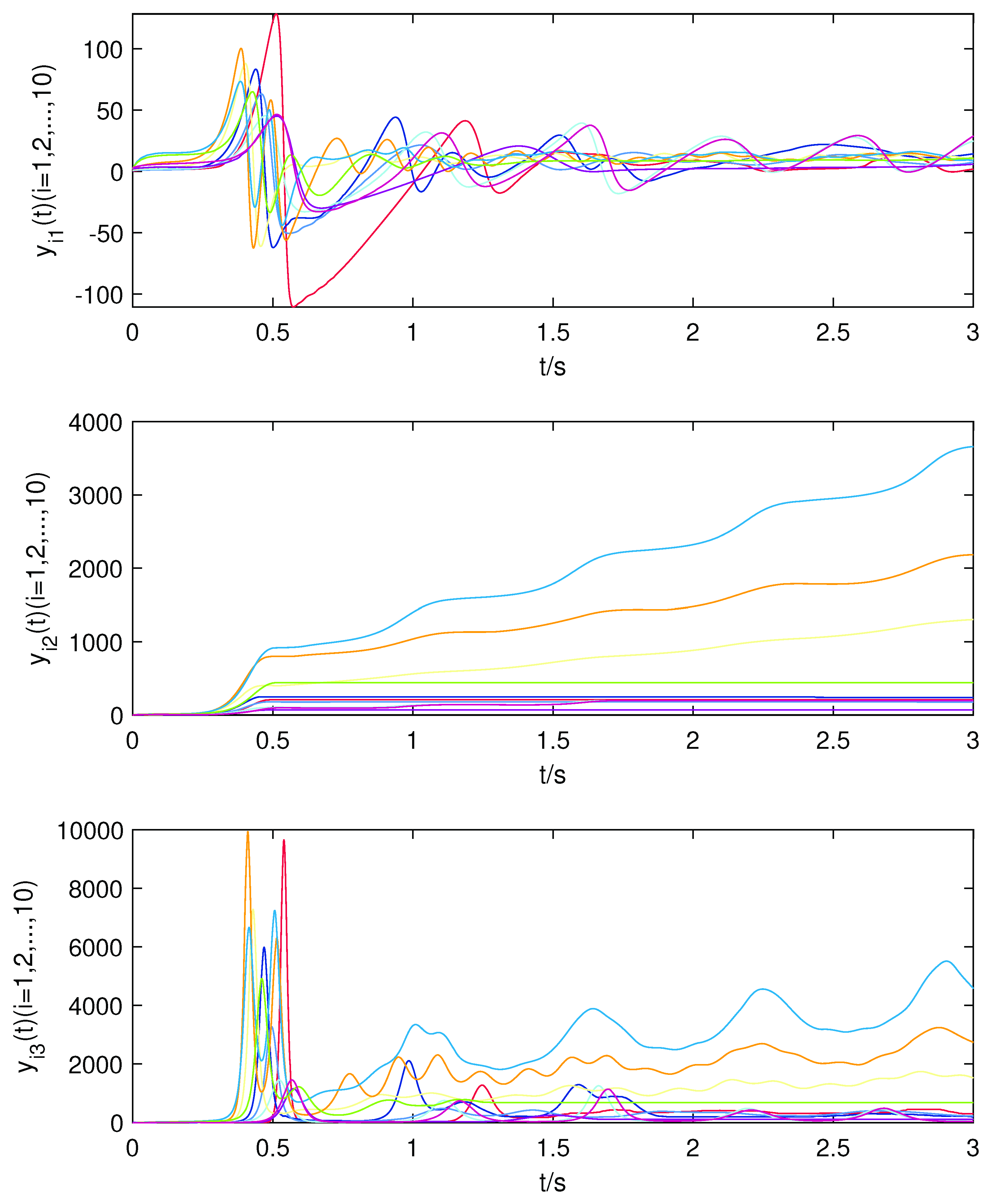

Supplement: S9 Fig — (TIFF) [file pone.0337899.s009.tif]

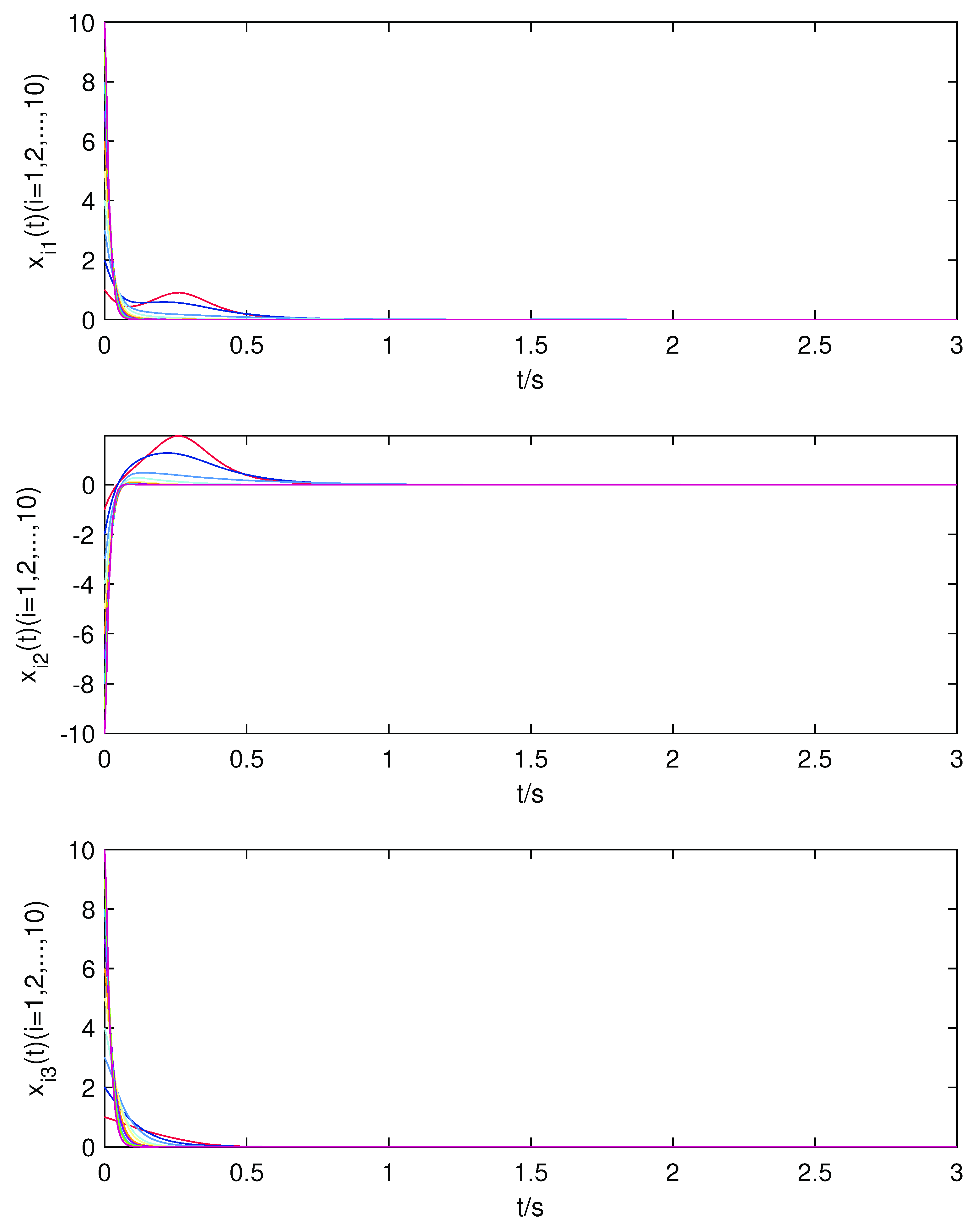

Supplement: S10 Fig — (TIFF) [file pone.0337899.s010.tif]

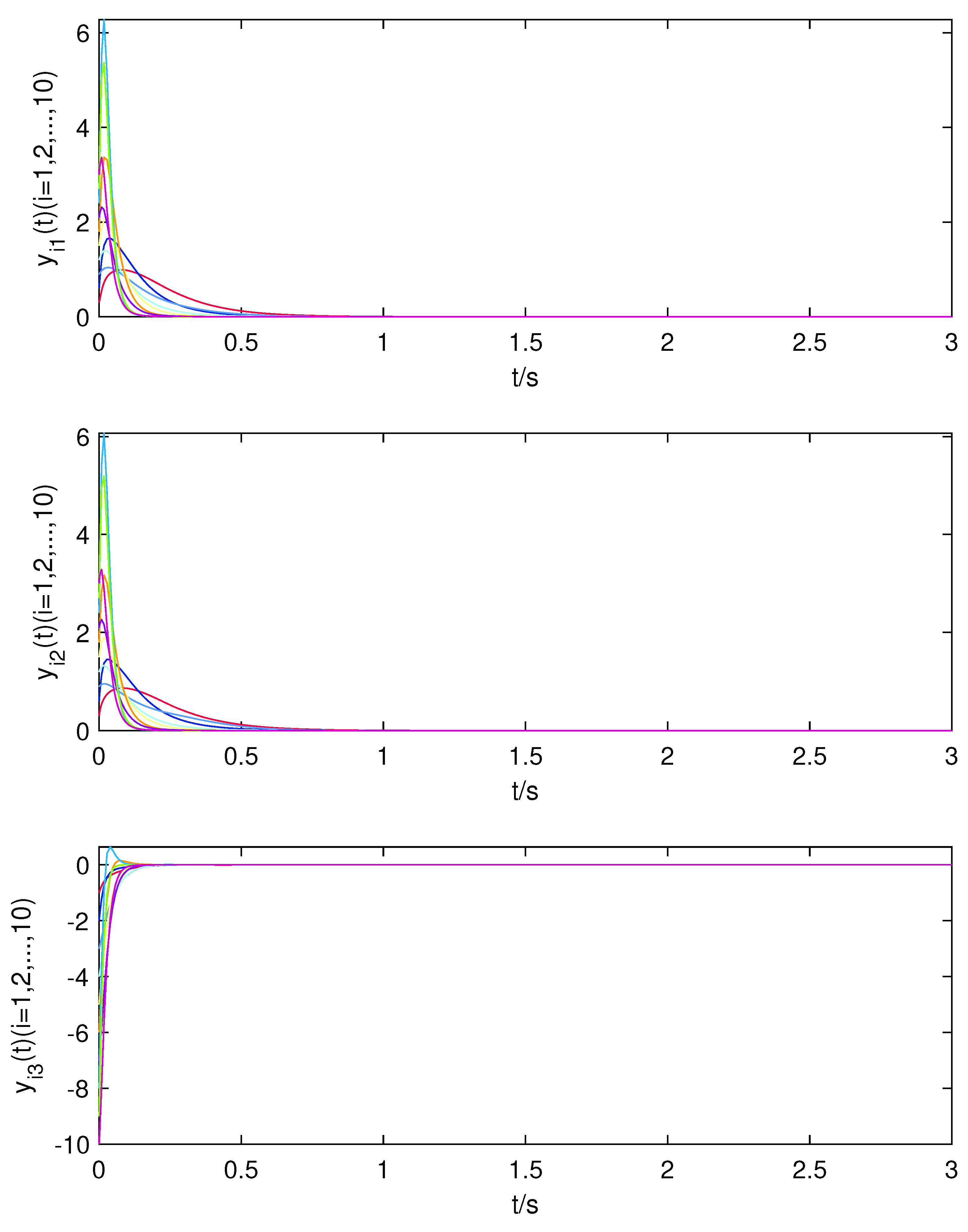

Supplement: S11 Fig — (TIFF) [file pone.0337899.s011.tif]

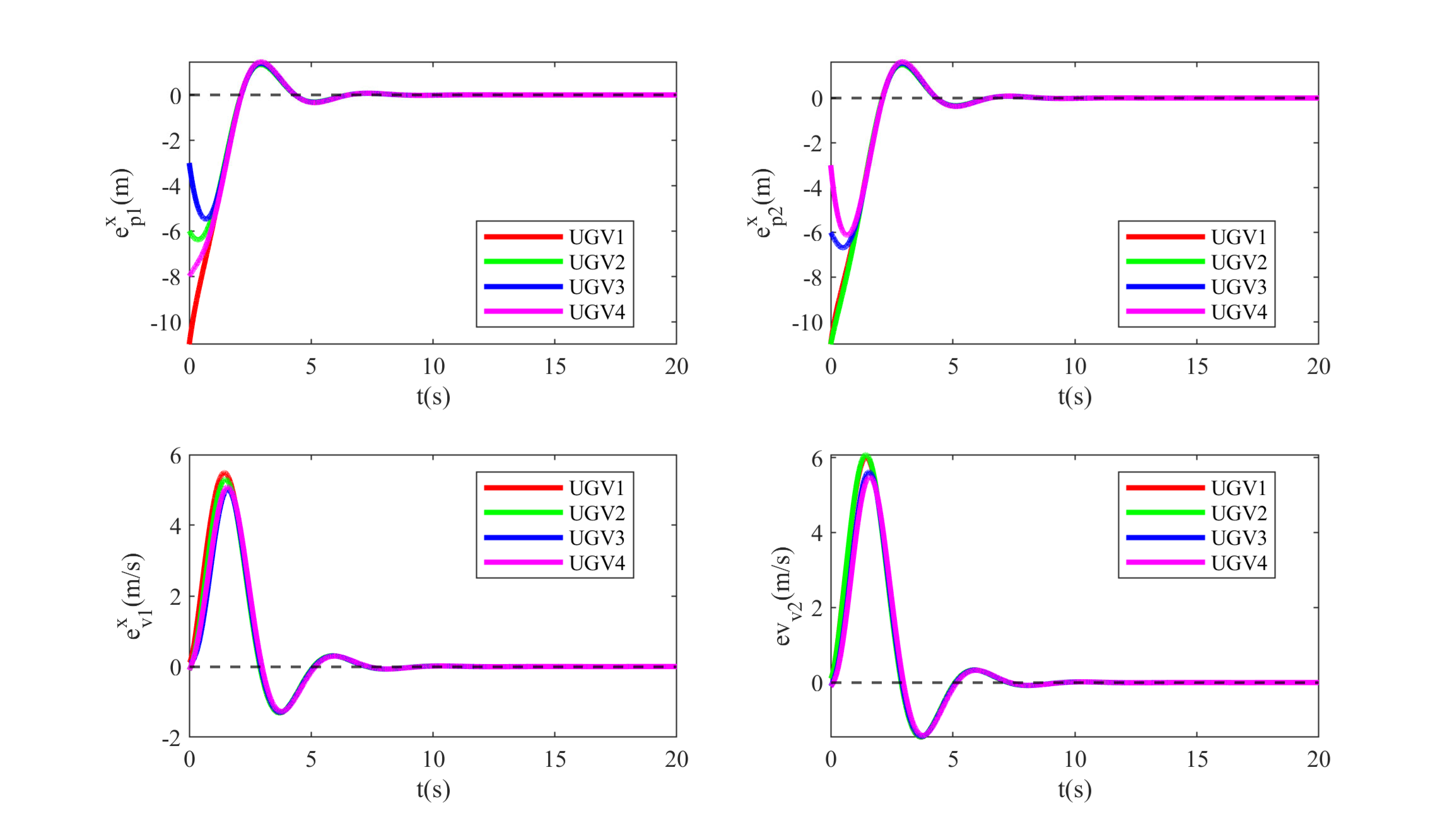

Supplement: S12 Fig — (TIFF) [file pone.0337899.s012.tif]

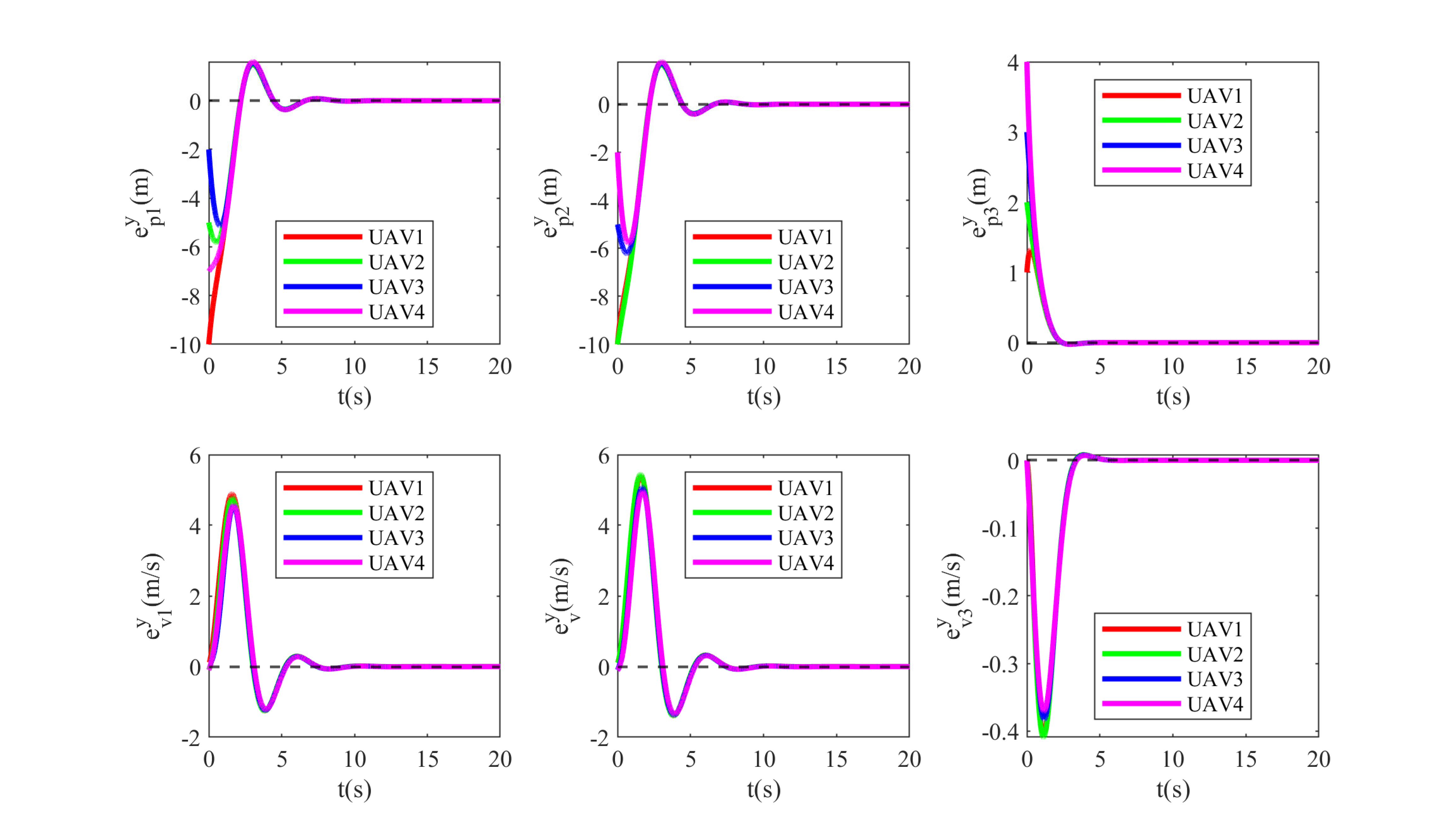

Supplement: S13 Fig — (TIFF) [file pone.0337899.s013.tif]

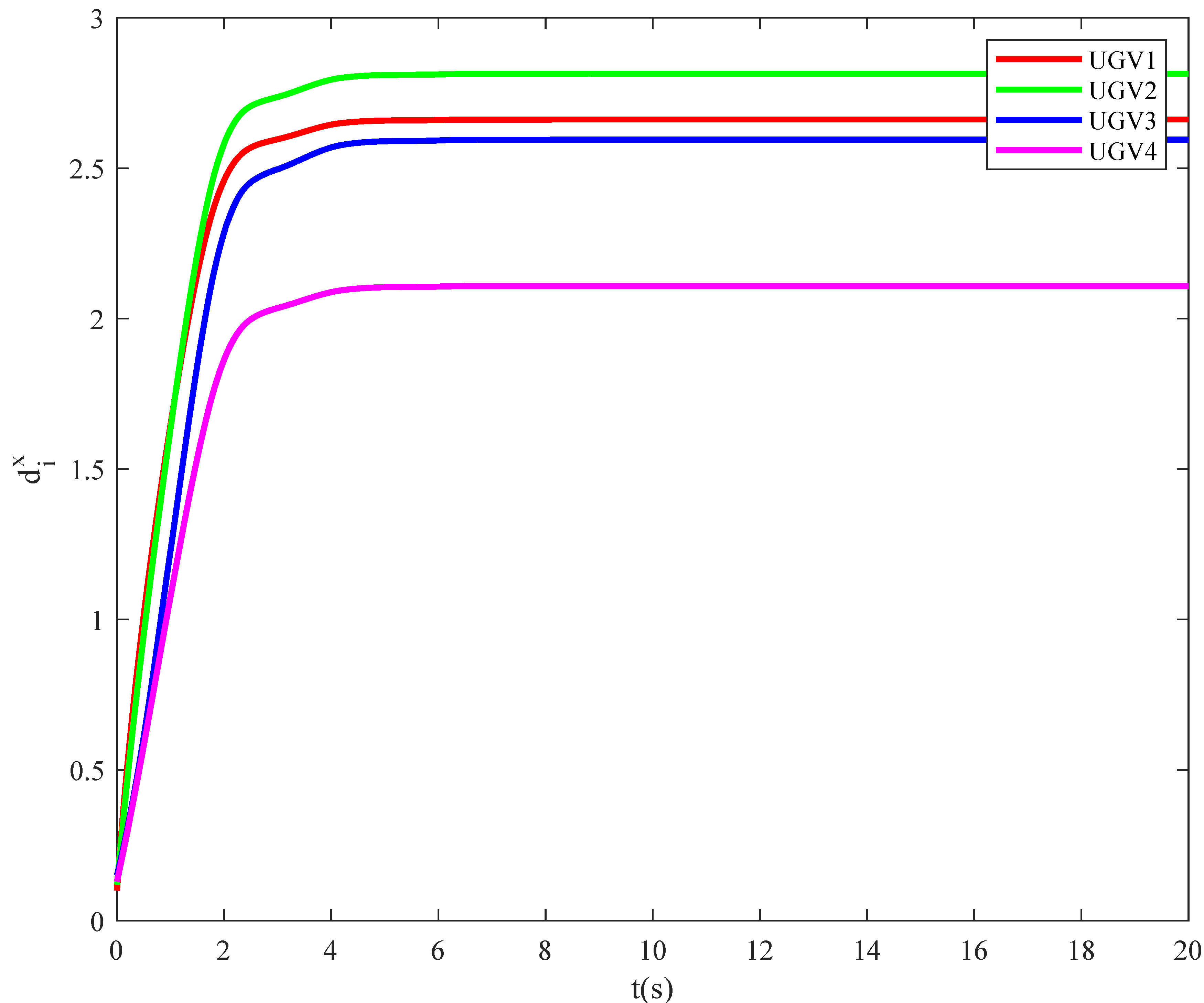

Supplement: S14 Fig — (TIFF) [file pone.0337899.s014.tif]

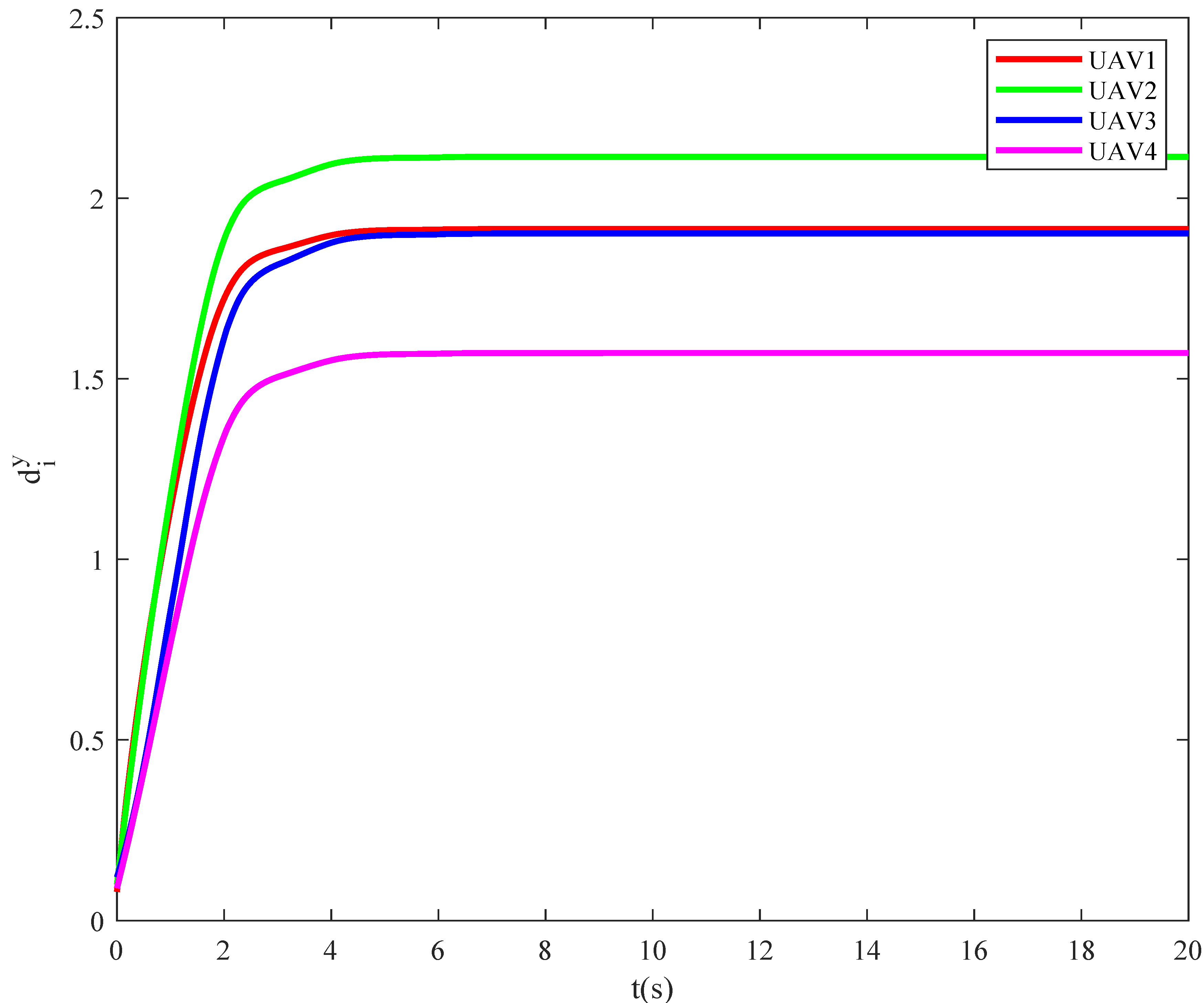

Supplement: S15 Fig — (TIFF) [file pone.0337899.s015.tif]
