# Supplementary material for: Stabilization of interdependent networks with two sub-networks of non-identical nodes
Source: PLoS One. 2025 Dec 26;20(12):e0337899. doi: 10.1371/journal.pone.0337899 (PMC12742803; doi:10.1371/journal.pone.0337899)
Supplement: Attachment — Submitted filename: Response to Reviewers.pdf [file pone.0337899.s016.pdf]

Response to Reviewer Comments

Paper No. : PONE-D-25-15972

Title: Stabilization of interdependent networks with two sub-networks of non-identical nodes

Authors: Cao Chen, Changyuan Guo, Tong Wang

Response to Editor:

[General Comment]:

Thank you for submitting your manuscript to PLOS ONE. After careful consideration, we feel that it has merit but does not fully meet PLOS ONE's publication criteria as it currently stands. Therefore, we invite you to submit a revised version of the manuscript that addresses the points raised during the review process.

[Reply]:

We would like to sincerely thank you for handling our manuscript entitled "Stabilization of interdependent networks with two sub-networks of non-identical nodes". We appreciate your recognition that the manuscript has merit, and we fully understand your concern that, in its current form, it does not yet fully meet the publication criteria of PLOS ONE.

In the revised version, we have carefully addressed the issues raised during the review process. We believe that these revisions improve the manuscript and bring it in line with the publication criteria of PLOS ONE. We are sincerely grateful for your constructive feedback and for providing us with the opportunity to strengthen our work.

[Additional Comment]:

Please find mind and the referee report in the message below. My main concern is whether this paper has enough material to be considered an original contribution. Please elaborate on this carefully.

The central claim of the manuscript concerning stabilization of interdependent networks with non-identical nodes is essentially a straightforward extension of well-known control schemes. The "non-identical nodes" angle plays only a minor role: the proof just reuses standard Lipschitz-type assumptions and does not exploit any deeper structure. This makes the contribution marginal. The validation is also weak with toy numerical examples are shown. Both are deterministic toy models. There is no exploration of robustness, scalability, parameter sensitivity, or relevance to real interdependent networks. The paper claims importance for "transportation systems, power grids, communication networks," but there is zero link to reality. This gap between the application claims and the toy theoretical exercise makes the paper feel disconnected and overstated.

[Reply]:

Thank you for your time and effort in handling the review process. We extend our sincere gratitude to the editor and reviewers for providing constructive comments that have greatly helped us enhance the quality of our paper. We have carefully revised the manuscript to clarify our contributions and strengthen the validation.

First, our study differs fundamentally from existing works [17,24,28,32], which primarily investigate homogeneous interdependent networks composed of nodes with identical dynamics. In contrast, real-world systems are typically heterogeneous. For example, in vehicle platoons, individual vehicles follow

distinct nonlinear dynamics because they have different masses and time constants [40], [41]. Moreover, according to the models in [45], such heterogeneous vehicle dynamics also satisfy QUAD conditions, as explained in the newly added Remark 4 in response to Reviewer #1's Comment 1, which aligns with the assumptions adopted in our paper. To clarify this point, we have revised the Introduction in the manuscript as follows:

"One common assumption adopted by most of the previous works [16,17,24,28,32] is that the two sub-networks contain the same number of nodes, and in particular, the studies [17,24,28,32] further assume that all nodes in each sub-network follow identical dynamics. However, such assumptions are not always consistent with reality. In many real-world systems, the subsystems are heterogeneous. For instance, vehicle platoon systems usually consist of vehicles with heterogeneous dynamics, which, as shown in [40], [41], can be readily verified to satisfy QUAD conditions. Motivated by these practical settings, this work focuses on the stabilization of heterogeneous interdependent networks without imposing restrictions on identical node numbers or homogeneous dynamics. Removing these limitations fundamentally changes the dynamics of interdependent networks and may cause the failure of most previously proposed control strategies."

Second, we have elaborated on the contributions of this work in the revised Introduction. Specifically, the paper develops a stabilization framework for interdependent networks with heterogeneous nodes, thereby extending classical approaches that rely on homogeneous dynamics. We show that our controller accommodates heterogeneity without requiring identical structures, and we provide new Lyapunov-based techniques to establish stability under these conditions. This makes the contribution a meaningful theoretical advancement rather than a trivial extension. To make this contribution clearer, we have revised the statement of contributions as follows:

"The mathematical model of interdependent networks with two sub-networks is proposed. In contrast to previous works [17,24,28,32], where the sub-networks are assumed to contain the same number of identical nodes, our model allows the two sub-networks to have different numbers of nodes as well as different node dynamics. Moreover, the commonly imposed diffusive coupling condition on the outer coupling matrix is not required in this paper."

Third, we would like to clarify that the proposed control scheme is **scalable**. According to the model formulation and proof process, the results do not depend on a fixed number of nodes, which means that the two sub-networks can be extended to larger sizes while still preserving stability properties. To illustrate the practical relevance of this feature, we have added a new simulation example involving a UAV–UGV (Unmanned Aerial Vehicle–Unmanned Ground Vehicle) formation. This scenario reflects the type of heterogeneous interdependent systems frequently encountered in practice, where aerial and ground vehicles follow different nonlinear dynamics and the control objective is to assemble at designated positions while achieving a prescribed formation pattern. The simulation results, now included in Section IV, demonstrate the applicability of our approach to realistic heterogeneous systems.

To highlight this improvement, we have added the following description in Section IV:

"To illustrate the applicability of the proposed stabilization strategy, we consider a task of UAV–UGV (Unmanned Aerial Vehicle–Unmanned Ground Vehicle) formation control. In this example, the UAVs and UGVs are modeled with different nonlinear dynamics, representing a heterogeneous interdependent system. The control objective is to achieve formation rendezvous where both UGVs and UAVs maintain square formations with 2m spacing while moving toward the designated target location $p_d^x = [10,10]^T$ m

for UGVs and $p_d^y = [10, 10, 2]^T$ m for UAVs, with UAVs maintaining a constant height of 2m above the corresponding UGVs. Moreover, the final velocities should converge to zero, i.e., $v_d^x = 0_2, v_d^y = 0_3$, ensuring that the formation reaches a stable equilibrium at the target. Referring to [49], the UAV–UGV group is composed of 4 UAVs and 4 UGVs, and their dynamic models are given as follows:

$$\dot{x}_i = D_{xi} x_i + \sum_{j=1}^{N_x} a_{ij} H_x\left(x_i\right) x_j + E_{xi} u_i^x$$

$$\dot{y}_i = D_{yi} y_i + \sum_{j=1}^{N_y} b_{ij} H_y\left(y_i\right) y_j + \sum_{j=1}^{N_x} c_{ij} H_{xy}\left(y_i\right) x_j + E_{yi} u_i^y$$

where $x_i \in \mathbb{R}^4$ represents the state vector of the i-th UGV containing position and velocity components $[p_{1i}^x, p_{2i}^x, v_{1i}^x, v_{2i}^x]^T$, $y_i \in \mathbb{R}^6$ denotes the state vector of the i-th UAV with components $[p_{1i}^y, p_{2i}^y, p_{3_i}^y, v_{1i}^y, v_{2i}^y, v_{3i}^y]^T$, $D_{xi} \in \mathbb{R}^{4\times4}$ and $D_{yi} \in \mathbb{R}^{6\times6}$ are the system dynamics matrices for UGV and UAV respectively, $E_{xi} \in \mathbb{R}^{4\times2}$ and $E_{yi} \in \mathbb{R}^{6\times3}$ are the control input matrices.

In simulation, the communication topology matrices are:

$$A = B = \begin{bmatrix} 0 & 1 & 0 & 1 \\ 1 & 0 & 1 & 0 \\ 0 & 1 & 0 & 1 \\ 1 & 0 & 1 & 0 \end{bmatrix}, \quad C = \begin{bmatrix} 1 & 0 & 0 & 0 \\ 0 & 1 & 0 & 0 \\ 0 & 0 & 1 & 0 \\ 0 & 0 & 0 & 1 \end{bmatrix}$$

The system dynamics matrices for UGV i are:

$$D_{xi} = \begin{bmatrix} 0 & 0 & 1 & 0 \\ 0 & 0 & 0 & 1 \\ 0 & 0 & -\alpha_i & 0 \\ 0 & 0 & 0 & -\alpha_i \end{bmatrix}, \quad E_{xi} = \begin{bmatrix} 0 & 0 \\ 0 & 0 \\ 1 & 0 \\ 0 & 1 \end{bmatrix}$$

where $\alpha_i = 0.05 + 0.01i$ represents heterogeneous damping coefficients.

The system dynamics matrices for UAV i are:

$$D_{yi} = \begin{bmatrix} 0 & 0 & 0 & 1 & 0 & 0 \\ 0 & 0 & 0 & 0 & 1 & 0 \\ 0 & 0 & 0 & 0 & 0 & 1 \\ 0 & 0 & 0 & -\beta_i & 0 & 0 \\ 0 & 0 & 0 & 0 & -\beta_i & 0 \\ 0 & 0 & 0 & 0 & 0 & -\beta_i \end{bmatrix}, \quad E_{yi} = \begin{bmatrix} 0 & 0 & 0 \\ 0 & 0 & 0 \\ 0 & 0 & 0 \\ 1 & 0 & 0 \\ 0 & 1 & 0 \\ 0 & 0 & 1 \end{bmatrix}$$

Where $\beta_i = 0.08 + 0.02i$.

The desired formation vectors define the relative positions of each agent with respect to the formation center:

$$w_1 = \begin{bmatrix} 1 \\ 1 \end{bmatrix}, \quad w_2 = \begin{bmatrix} -1 \\ 1 \end{bmatrix}, \quad w_3 = \begin{bmatrix} -1 \\ -1 \end{bmatrix}, \quad w_4 = \begin{bmatrix} 1 \\ -1 \end{bmatrix}$$

$$r_1 = \begin{bmatrix} 1 \\ 1 \\ 0 \end{bmatrix}, \quad r_2 = \begin{bmatrix} -1 \\ 1 \\ 0 \end{bmatrix}, \quad r_3 = \begin{bmatrix} -1 \\ -1 \\ 0 \end{bmatrix}, \quad r_4 = \begin{bmatrix} 1 \\ -1 \\ 0 \end{bmatrix}$$

Correspondingly, the tracking errors for UGVs are defined as:

$$e_p^x = p^x - (p_d^x + w_i)$$

$$e_v^x = v^x - 0$$

The tracking errors for UAVs are defined as:

$$e_p^y = p^y - (p_d^y + r_i)$$

$$e_v^y = v^y - 0$$

According to (8) and (9), the adaptive control laws are designed as:

$$u_i^x = -d_i^x(e_{pi}^x + e_{vi}^x)$$

$$u_i^y = -d_i^y(e_{pi}^y + e_{vi}^y)$$

To maintain the formation with UAVs maintaining a constant height of 2m above the corresponding UGVs, coupling terms are:

$$H_x(x_i)x_j = [0, 0, e_p^x + e_v^x]^T$$

$$H_y(y_i)y_j = [0, 0, 0, e_p^y + e_v^y]^T$$

$$H_{xy}(y_i)x_j = [0, 0, 0, -(p_{1i}^y - p_{1j}^x) - (v_{1i}^y - v_{1j}^x), -(p_{2i}^y - p_{2j}^x) - (v_{2i}^y - v_{2j}^x), -(p_{3i}^y - 2) - v_{3i}^y]^T$$

The initial conditions are set as: $x_1(0) = [0, 0, 0.1, 0.1]^T$, $x_2(0) = [3, 0, -0.1, 0.1]^T$, $x_3(0) = [6, 3, 0.1, -0.1]^T$, $x_4(0) = [3, 6, -0.1, -0.1]^T$, $y_1(0) = [1, 1, 3, 0.1, 0.1, 0]^T$, $y_2(0) = [4, 1, 4, -0.1, 0.1, 0]^T$, $y_3(0) = [7, 4, 5, 0.1, -0.1, 0]^T$, $y_4(0) = [4, 7, 6, -0.1, -0.1, 0]^T$ with initial adaptive gains $d_i^x(0) = [0.1, 0.12, 0.15, 0.13]^T$ and $d_i^y(0) = [0.08, 0.1, 0.12, 0.09]^T$.

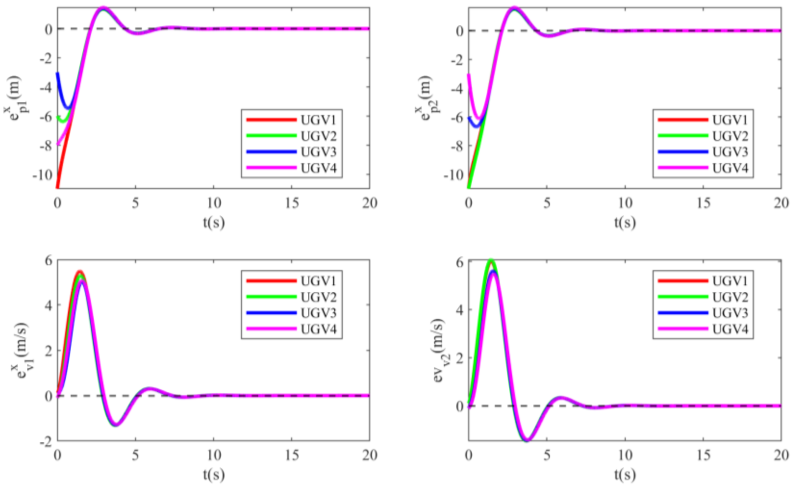

Fig. 12. The tracking errors of the UGVs.

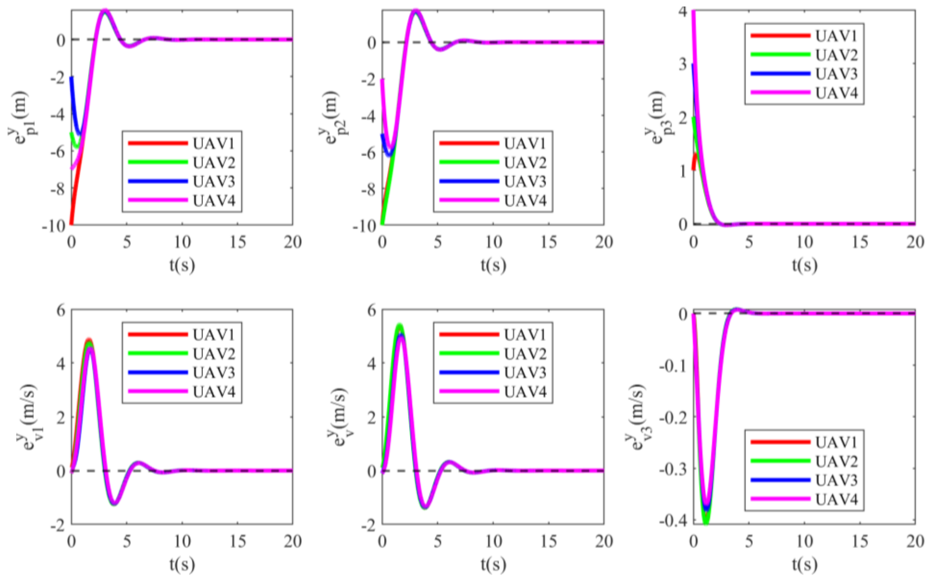

Fig. 13. The tracking errors of the UAVs.

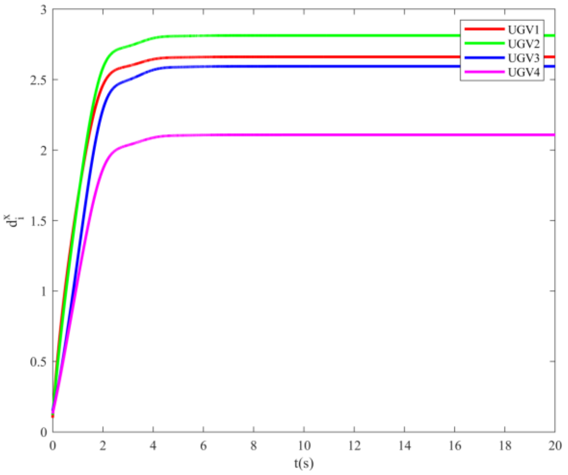

Fig. 14. The evolution of the d_i^x of UGVs.

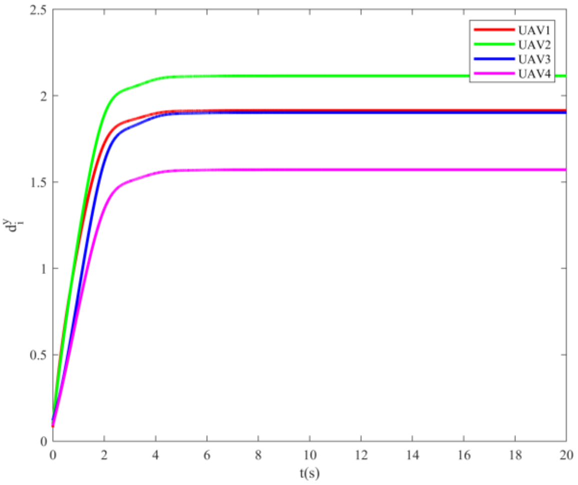

Fig. 15. The evolution of the d_i^y of UAVs.

Simulation results are depicted in Figs 12-15. Fig. 12 shows the tracking errors of the UGVs, which converge asymptotically to zero. Fig. 13 presents the tracking errors of the UAVs, which also vanish over time. Figs. 14 and 15 illustrate the adaptive laws of the controller parameters for the UGVs and UAVs, respectively, where all adaptive parameters remain bounded.

In summary, the simulation results demonstrate that the proposed control law successfully achieves the UAV–UGV formation control objectives and exhibits practical applicability."

The newly added reference in the revised paper is given below:

40. Y. Zhu et al., Joint sliding-mode controller and observer for vehicle platoon subject to disturbance and acceleration failure of neighboring vehicles, IEEE Transactions on Intelligent Vehicles. 2023; 8(3): 2345-2357, March

41. Y. Li et al., Variable time headway policy based platoon control for heterogeneous connected vehicles with external disturbances, IEEE Transactions on Intelligent Transportation Systems. 2022; 23(11): 21190-21200.

49. Tang H, Chen Y, Ali I. Cross-dimensional distributed control for heterogeneous UAV-UGV systems with nonzero leader input. IEEE Transactions on Intelligent Vehicles. 2024; 9(10): 6354-6368.

Response to Reviewer #1

[General Comments]:

As a whole the manuscript "Stabilization of interdependent networks with two sub-networks of non-identical nodes" is interesting and could be published on PLOS ONE, but before that a few points need to be addressed.

[Reply]:

Thank you for your comments. We appreciate your constructive comments and make the following responses to address your concerns.

[Comment 1]:

In the introduction the authors insist on the importance of having sub-networks with different nodes. But when they present their model, see eq. 1 and 2 are very similar. In particular the authors are not clear whether or not the functions f and g are equal or just represent the same (in this case dynamics) in the equations. This is crucial to the paper. Please explain.

[Reply]:

We would like to express our sincere gratitude to Reviewer 1 for the careful reading of our manuscript "Stabilization of interdependent networks with two sub-networks of non-identical nodes" and for recognizing its potential interest for publication in PLOS ONE. We truly appreciate your encouraging remark that the manuscript is interesting and could be published after revisions.

In response, we have carefully revised the manuscript and provide the following detailed replies to address all the issues you raised.

[Comment 2]:

Assumption 1: what is N? Are both sub-networks equal?
Please, explain the meaning of assumption 2 (eq 3) and assumption 3 (eq 4).

[Reply]:

Thank you for your valuable comments. We appreciate your constructive comments and make the following responses to address your concerns.

Regarding Assumption 1

We acknowledge that the original statement in Assumption 1 was not precise. Specifically, we mistakenly wrote "for any given state vector $x_i, y_i, i=1,\ldots,N$," which could misleadingly suggest that both sub-networks contain the same number of nodes. In fact, as defined in the model section, the two sub-networks have N_x and N_y nodes, respectively, and in general $N_x \neq N_y$.

To correct this ambiguity, we have revised Assumption 1 as follows:

"For any given state vectors $x_i \in \mathbb{R}^n (i=1,\ldots,N_x)$ and $y_i \in \mathbb{R}^n (i=1,\ldots,N_y)$, there exists a positive constant $m>0$ such that $\| H_x(x_i) \| \le m$, $\| H_y(y_i) \| \le m$, and $\| H_{xy}(y_i) \| \le m$."

In addition, we also have revised Remark 2 before Assumption 1 to explicitly state the model formulation as follows:

" N_x and N_y denote the numbers of nodes in sub-networks X and Y, respectively; the two sub-networks do not need to have the same size, and their nodes are not required to be identical."

Regarding Assumptions 1 and 2

These two assumptions are based on the well-known QUAD (Quadratic) condition [44], which is a standard assumption in the study of synchronization and stability of nonlinear complex networks[R1]-[R3]. A nonlinear vector field $f:\mathbb{R}^n \to \mathbb{R}^n$ is said to be QUAD (Δ, ω), if for any vector $\xi_1, \xi_2 \in \mathbb{R}^n$,

$$(\xi_1-\xi_2)^{\mathrm{T}}[f(\xi_1)-f(\xi_2)]-(\xi_1-\xi_2)^{\mathrm{T}}\Delta(\xi_1-\xi_2) \le -\omega(\xi_1-\xi_2)^{\mathrm{T}}(\xi_1-\xi_2)$$

where Δ is a diagonal matrix and ω is a real positive scalar.

This condition is widely used in the literature, because they ensure that the growth rate of the nonlinear term $f(\cdot)$ is quadratically bounded by the distance between ξ_1 and ξ_2. In other words, it is a generalized Lipschitz condition. The QUAD condition is not only a technical tool but also has clear physical meaning: many real-world systems exhibit nonlinear dynamics that naturally satisfy such quadratic constraints. For example, models of neural oscillators, power system dynamics, and even chaotic systems like the Lorenz oscillator have been shown to satisfy QUAD-type inequalities [45].

In our manuscript, Assumption 2 (Eq. 3) and Assumption 3 (Eq. 4) correspond precisely to applying the QUAD $((\gamma_i+\omega)I_n, \omega)$ and QUAD $((\beta_i+\omega)I_n, \omega)$ conditions, respectively, to the node dynamics of sub-networks *X* and *Y*.

To address the reviewer's concern, we have added a new Remark 4 to explain the meaning of Assumptions 2 and 3 as follows:

"**Remark 4.** Assumptions 2 and 3 are derived from the QUAD (Quadratic) condition, which generalizes the Lipschitz property for nonlinear systems [44]. These assumptions ensure that the growth rate of the nonlinearities of both sub-networks are quadratically bounded. Since many real-world systems (e.g., power systems and chaotic systems) naturally satisfy QUAD conditions [28], [45], these assumptions are practically reasonable."

The newly added reference in the revised paper is given below:

44. DeLellis P, di Bernardo M. Russo G. On QUAD, Lipschitz, and contracting vector fields for consensus and synchronization of networks IEEE Transactions on Circuits & Systems I Regular Papers. 2011; 58(3):576-583.
45. DeLellis P, Garofalo F. Novel decentralized adaptive strategies for the synchronization of complex networks. Automatica, 2009, 45(5): 1312-1318.

Reference

[R1] Yang X, Lu J. Finite-time synchronization of coupled networks with Markovian topology and impulsive effects. IEEE Transactions on Automatic Control, 2015, 61(8): 2256-2261.

[R2] Burbano-L D A, Russo G, Di Bernardo M. Pinning controllability of complex network systems with noise. IEEE Transactions on Control of Network Systems, 2018, 6(2): 874-883.

[R3] Dai M Z, Ahn C K, Zhang C, et al. On prescribed performance synchronization to QUAD nonlinear multi-agent networks. IEEE Transactions on Circuits and Systems II: Express Briefs, 2021, 69(3): 1377-1381.
