# Supplementary material for: Stabilization of interdependent networks with two sub-networks of non-identical nodes
Source: PLoS One. 2025 Dec 26;20(12):e0337899. doi: 10.1371/journal.pone.0337899 (PMC12742803; doi:10.1371/journal.pone.0337899)
Supplement: Attachment — Submitted filename: Response to Reviewers.docx [file pone.0337899.s017.docx]

**Response to Reviewer Comments**

**Paper No.** **:** PONE-D-25-15972R1

**Title:** Stabilization of interdependent networks with two sub-networks of non-identical nodes

**Authors:** Cao Chen, Changyuan Guo, Tong Wang

**Response to Editor:**

[General Comment]:

Thank you for submitting your manuscript to PLOS ONE. After careful consideration, the referee still has suggestions to improve your manuscript. Therefore, we invite you to submit a revised version of the manuscript that addresses the points raised during the review process.

[Reply]:

Thank you for your time and effort in handling the review process. We extend our gratitude to the editor and reviewers for providing constructive comments that have helped us significantly enhance the quality of our paper. We have carefully reviewed and addressed all the feedback and have made revisions to the manuscript accordingly.

We believe that these revisions comprehensively address the reviewer’s concerns and significantly enhance the quality of our work. We are sincerely grateful for your constructive feedback and for providing us with the opportunity to strengthen our work.

**Response to Reviewer #1**

[General Comments]:

I am satisfied with the authors responses. I think that now the manuscript can be considered for publication in PLOS ONE, after correcting a few minor problems, which I point below.

[Reply]:

Thank you for your kind comments. We appreciate your constructive comments and make the following responses to address your concerns.

[Comment 1]:

Introduction: 4th paragraph: Explain an acronym (QUAD) the first time it appears in the manuscript.

[Reply]:

Thank you for your valuable comments. Based on your suggestion, we have revised corresponding sentence as follows:

“For instance, vehicle platoon systems usually consist of vehicles with heterogeneous dynamics, which, as shown in [40,41], can be readily verified to satisfy quadratic (QUAD) conditions.”

[Comment 2]:

Preliminaries and model formulations: Check Capital letters!

[Reply]:

Thank you for your valuable comments. The capitalization of “Preliminaries and model formulations” has been corrected. In addition, we have carefully checked all other section titles to ensure consistency in capitalization throughout the manuscript.

[Comment 3]:

Example 3.

Before equation (26) the authors mention: the UAV–UGV group is composed of 4 UAVs and 4 UGVs, and their dynamic models are given as follows: But later they say: Dxi ∈ R4×4 and Dyi ∈ R6×6, which is clear in eqs. 29 and 30. Correct the phrase before Eq. 26!

[Reply]:

We agree that the original description “the UAV–UGV group is composed of 4 UAVs and 4 UGVs” may cause ambiguity, as it could be interpreted that both UAVs and UGVs share identical state dimensions. In fact, the UGV operates in a 2D plane, and its state vector, while the UAV operates in 3D space with. Correspondingly, the system matrices for UGV and UAV are and, respectively. To avoid misunderstanding, we have revised the sentence before Eq. (26) to read as:

“the UAV–UGV group is composed of 4 UGVs (operating in 2D) and 4 UAVs (operating in 3D)”

This revision clearly distinguishes the dimensionality of each subsystem and ensures consistency with Eqs. (26)-(27).
